# Isolation and Characterization of a Virulent Bacteriophage AB1 of *Acinetobacter baumannii*

**DOI:** 10.1186/1471-2180-10-131

**Published:** 2010-04-29

**Authors:** Hongjiang Yang, Li Liang, Shuxiang Lin, Shiru Jia

**Affiliations:** 1 Key Laboratory of Industrial Microbiology, Ministry of Education, PO Box 08, Tianjin University of Science & Technology, TEDA, Tianjin 300457, PR China; 2 Tianjin Children's Hospital, 225 Machang Road, Tianjin 300074, PR China

## Abstract

**Background:**

*Acinetobacter baumannii *is an emerging nosocomial pathogen worldwide with increasing prevalence of multi-drug and pan-drug resistance. *A. baumannii *exists widely in natural environment, especially in health care settings, and has been shown difficult to be eradicated. Bacteriophages are often considered alternative agent for controlling bacterial infection and contamination. In this study, we described the isolation and characterization of one virulent bacteriophage AB1 capable of specifically infecting *A. baumannii*.

**Results:**

A virulent bacteriophage AB1, specific for infecting a clinical strain *A. baumannii *KD311, was first isolated from marine sediment sample. Restriction analysis indicated that phage AB1 was a dsDNA virus with an approximate genome size of 45.2 kb to 46.9 kb. Transmission electron microscopy showed that phage AB1 had an icosahedral head with a non-contractile tail and collar or whisker structures, and might be tentatively classified as a member of the *Siphoviridae *family. Proteomic pattern of phage AB1, generated by SDS-PAGE using purified phage particles, revealed five major bands and six minor bands with molecular weight ranging from 14 to 80 kilo-dalton. Also determined was the adsorption rate of phage AB1 to the host bacterium, which was significantly enhanced by addition of 10 mM CaCl_2_. In a single step growth test, phage AB1 was shown having a latent period of 18 minutes and a burst size of 409. Moreover, pH and thermal stability of phage AB1 were also investigated. At the optimal pH 6.0, 73.2% of phages survived after 60 min incubation at 50°C. When phage AB1 was used to infect four additional clinical isolates of *A. baumannii*, one clinical isolate of *Stenotrophomonas maltophilia*, and *Pseudomonas aeruginosa *lab strains PAK and PAO1, none of the tested strains was found susceptible, indicating a relatively narrow host range for phage AB1.

**Conclusion:**

Phage AB1 was capable of eliciting efficient lysis of *A. baumannii*, revealing its potential as a non-toxic sanitizer for controlling *A. baumannii *infection and contamination in both hospital and other public environments.

## Background

*Acinetobacter baumannii *is a nonfermentative, nonmotile, catalase-positive, gram-negative bacterium found in soil, water, sewage, and many health care environments. *A. baumannii *is also a commensal microbe existing on human skin and mucous membrane, capable of opportunistic infections, especially in immunocompromised individuals, including pneumonia, meningitis, septicaemia, and urinary tract infection [1,2]. Since its first discovery, *A. baumannii *has become resistant to many common antibiotics due to both intrinsic mechanisms and its capability to acquire drug resistance determinants. The increasing prevalence of multi-drug and pan-drug resistant *A. baumannii *strains found in clinics has rendered it one of the few important nosocomial pathogens, only next to *Pseudomonas aeruginosa *among non-fermentative gram-negative bacteria [3,4]. *A. baumannii *is resistant to dehydration, UV radiation, common chemical sanitizers, and detergents, making it extremely difficult to eradicate *A. baumannii *contaminations from hospital settings, especially catheter-related devices used in intensive care units (ICU). In fact, regular antimicrobial agents only inhibit its growth. Currently, there are no procedures available for removing *A. baumannii *in hospital environments, greatly increasing the risk of hospitalized patients, especially patients in ICU, to the infection by antibiotic-resistant *A. baumannii *[5,6].

Recently, there have been renewed interests in the researches and applications of bacteriophages as antibacterial agent, partly due to their specificity in targeting and lysing host bacteria [7-9]. Discovered over one hundred years ago, bacteriophages have been successfully used in the treatments of various infectious diseases. As an alternative to antibiotic therapy, bacteriophage therapy is potentially a powerful approach for the treatment of bacterial infection, especially when antibiotic resistance is increasingly becoming a serious challenge facing the medical community [10,11]. Recently, bacteriophage preparations have been approved by the Food and Drug Administration of USA as a food additive in ready-to-eat products to prevent foodborne bacterial diseases [12]. Animal tests of phage therapy are being conducted for treatments of various bacteria infections, and many lytic phages have been isolated and tested for such applications [13].

Phages specific to *Acinetobacter *genus were isolated and subsequently used in phage-typing of *Acinetobacter spp. *isolates [14,15]. Using an animal model, Soothill examined phage efficacy against infections caused by *A. baumannii*. Specifically, tested mice survived the otherwise lethal challenge of 5 LD50 (1 × 10^8^) cells of a virulent *A. baumannii *strain, when protected by as few as 10^2 ^PFU of one lytic *Acinetobacter *phage [16,17]. However, to our best knowledge, no detailed characterizations on any lytic *A. baumannii *phages have been reported [18,19]. In this paper, clinical isolates of *A. baumannii *were collected and used as indicator hosts for screening phages in marine sediment sample. Virulent phage AB1 was isolated and characterized. The results showed phage AB1 as a double-stranded DNA bacterial virus capable of efficiently lysing *A. baumannii *KD311.

## Results

### Identification of *A. baumannii *clinical strains

Before starting phage screening, clinically isolated *Acinetobacter spp. *strains were first confirmed the identity of the *A. baumannii *by using sequence information derived from their 16S rRNA gene. As described in Material and Methods, DNA fragment containing 16S rRNA gene from each clinical isolate was PCR-amplified and sequenced. The resulted sequences were deposited to GenBank and aligned to search for the most similar sequences. Five collected clinical strains (KD311, KD312, KD331, KD332, and KD334) were validated to be *A. baumannii *and KD335 was *Stenotrophomonas maltophilia*, one pathogen often isolated accompanying with *A. baumannii *infections.

### Bacteriophage isolation

Five *A. baumannii *clinical isolates were used as indicator strains for virulent bacteriophages screening from marine sediment samples. After enrichment, phage-containing samples were plated onto semi-solid agar plates with the indicator strain forming a bacterial lawn, and plaques were allowed to form by incubating at 35°C for 4 hours. Clear plaques were obtained from these samples only when strain KD311 served as the indicator, with plaques forming at size of about 1-2 mm in diameter. The phage isolate (named AB1) was selected for further study.

### Restriction fragment analysis of genomic DNA

**Figure 1:**
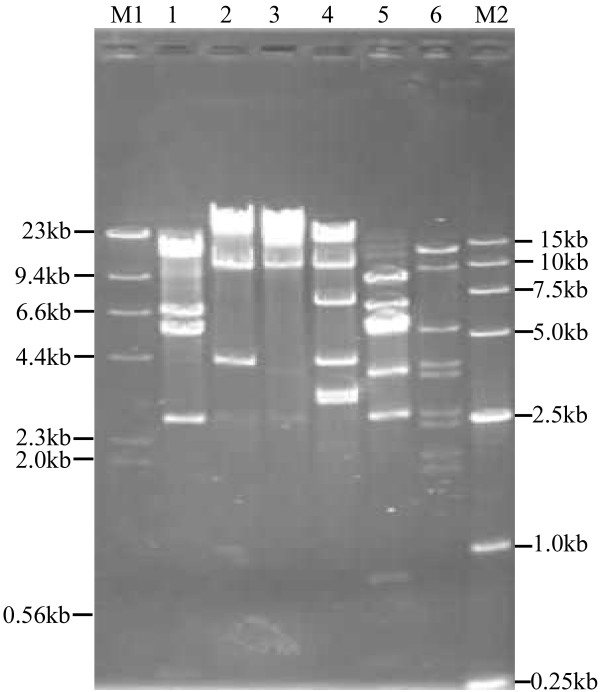
**Restriction fragments analysis of phage genomic DNA**. Phage genomic DNA was digested with *Eco*RI (lane1), *Xba*I (lane 2), *Bgl*II (lane 3), *Bgl*II/*Xba*I (lane 4), *Eco*RI/*Bgl*II (lane 5), and *Eco*RI/*Xba*I (lane 6), respectively. M1: molecular standard 1; M2: molecular standard 2.

Phage AB1 was amplified and its genomic DNA extracted as described. Purified genomic DNA was digested with several restriction endonucleases or their combiantions, including *Apa*I, *Bam*HI, *Bgl*II, *Eco*RI, *Eco*RV, *Hind*III, *Kpn*I, *Nco*I, *Pst*I, *Pvu*II, *Sal*I, *Sph*I, *Xba*I, *Bgl*II/*Xba*I, *Eco*RI/*Bgl*II, and *Eco*RI/*Xba*I, and subsequently subjected to electrophoretic analyses. As shown in Fig. 1, out of the tested enzymes, the enzyme combinations generated clear DNA patterns. Based on the digestion profiles of *Bgl*II/*Xba*I, *Eco*RI/*Bgl*II, and *Eco*RI/*Xba*I, the genome size was determined to be approximately at the range of 45.2 kb to 46.9 kb. The restriction analyses also indicated that phage AB1 was a dsDNA virus. Determination of the phage genome sequence is also underway.

### Morphology study by transmission electron microscopy

**Figure 2:**
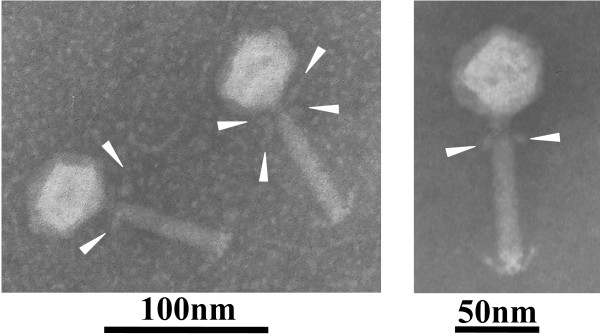
**Transmission electron micrograph of phage particles**. Virions were negatively stained with potassium phosphotungstate. The bar represents a length of 100 nm or 50 nm. Blank arrows indicate collar or whisker structure of phage AB1.

Phage AB1 solution was filtrated with amicon-100 filter to remove soluble macromolecules up to 100 KD in size. After washing three times with 0.1 M ammonium acetate solution, the retained phage solution was used directly for negative staining. Images of phage AB1 were developed using transmission electron microscope (Fig. 2). The results showed that phage AB1 had an icosahedral head, about 50 nm in diameter, a 80 nm long non-contractile tail, and collar or whisker structures, thus morphologically similar to phages belonging to *Siphoviridae *family.

### Proteomic analysis of phage structural proteins

**Figure 3:**
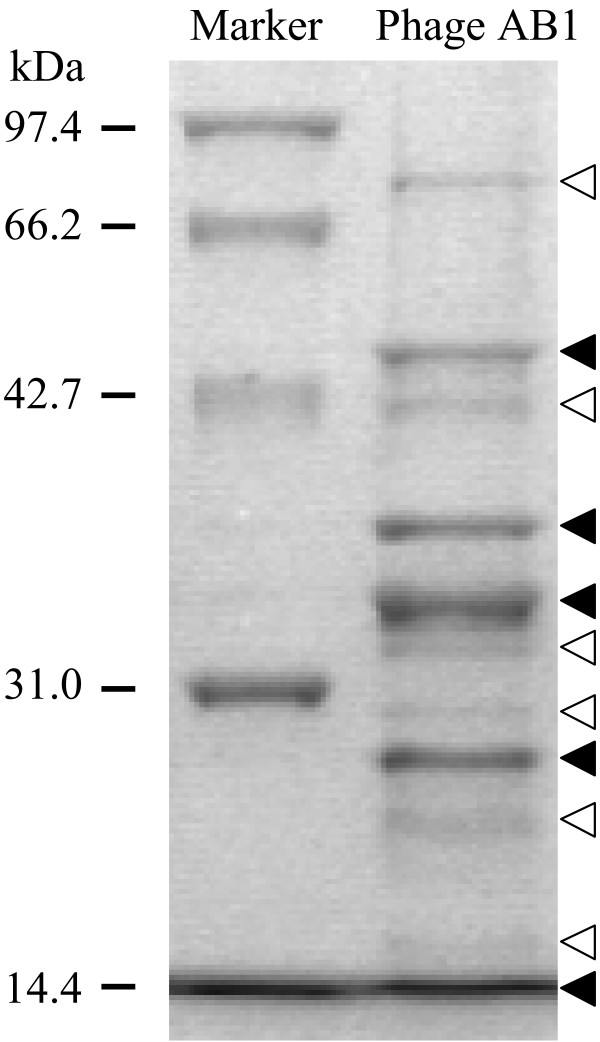
**SDS-PAGE analysis of phage structural proteins**. Phages particles from PEG precipitation was loaded directly. ▼: solid arrows indicate major proteins bands; ▽: blank arrows show minor proteins bands.

Purified phage particles were subjected to SDS-PAGE and proteomic patterns were obtained after Coomassie Blue G-250 staining and destaining (Fig. 3). Totally, five major protein bands and six minor protein bands were observed on the gel, with molecular weights ranging from 14 to 80 kilo-dalton.

### Determination of the multiplicity of infection (MOI)

*A. baumannii *culture of exponential growth phase was aliquot into vials with equal number of bacterial cells (10^8 ^cfu), which were infected with different amount of phage AB1 as designed, then plated after 4 hours of incubation. The group with a MOI of 10^-4 ^gave the highest production of phage progeny (4 × 10^10 ^PFU/ml), and the MOI of 10^-4 ^was chosen for the subsequent experiments in this study.

### Analysis of calcium effect on adsorption rate

**Figure 4:**
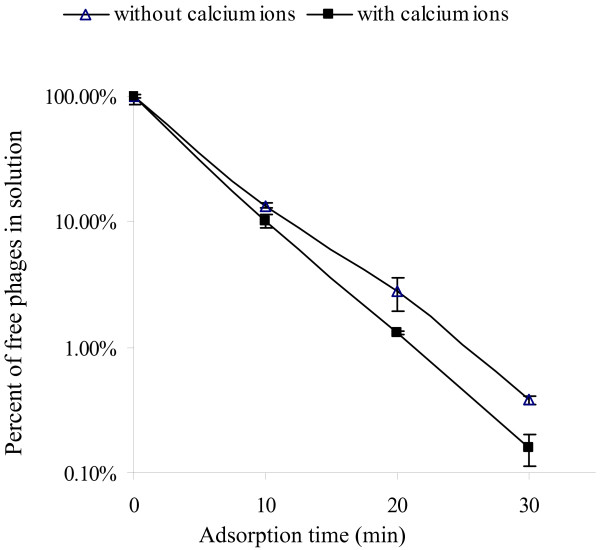
**Adsorption rate test**. At different time intervals, samples were taken from the supernatants to measure free phage particles. Divalent metal ions effect on adsorption rate was analyzed by adding 10 mM CaCl_2 _to the mixture of phage AB1 and *A. baumannii *cells.

Adsorption was the first step of phage infection of host bacteria and is often affected by the presence of divalent metal ions in the solution [20,21]. In the experiments, calcium ions were added to test their effects on adsorption efficacy. Phage AB1 and *A. baumannii *cells were mixed, free phage numbers, left in the solution, were detected at different time intervals. Statistical analysis showed significant differences existed between the two groups, and the results indicated calcium ions might stabilize phage adsorption process (Fig. 4).

### Latent time and phage burst size

**Figure 5:**
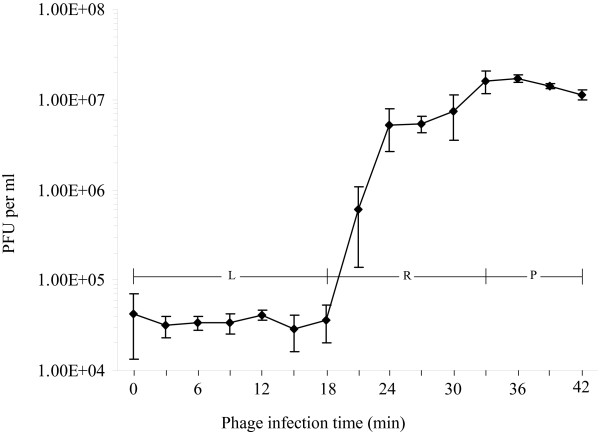
**One step growth experiment**. Latent time and burst size of phage AB1 were inferred from the curve with a triphasic pattern. L: latent phase; R: rise phase; P: plateau phase.

Single step growth experiment was performed to determine the latent time and phage burst size of phage AB1. As shown in Fig. 5, a triphasic curve, including the latent phase, rise phase, and plateau phase, was obtained. Using these data, the latent time was determined to be about 18 minutes, and the burst size of phage AB1 was 409 PFU/infected cell.

### pH and thermal stability tests

**Figure 6:**
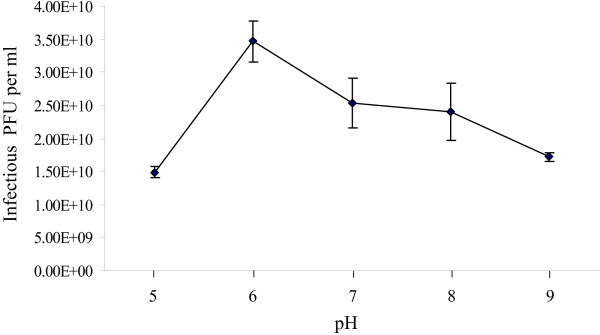
**pH stability test of phage AB1**. Phage was incubated under different pH values for one hour before determining the number of infectious phage particles.

Optimal pH was determined by testing the stability of phage AB1 under different pHs. Almost no reduction of infectious phage AB1 was observed after one hour incubation at pH6.0, while different reduction percentages were obtained at other pHs, only 42.9% recovery of infectious phage AB1 at pH5.0. These results suggested that extreme pHs might affect phage AB1 stability (Fig. 6).

**Figure 7:**
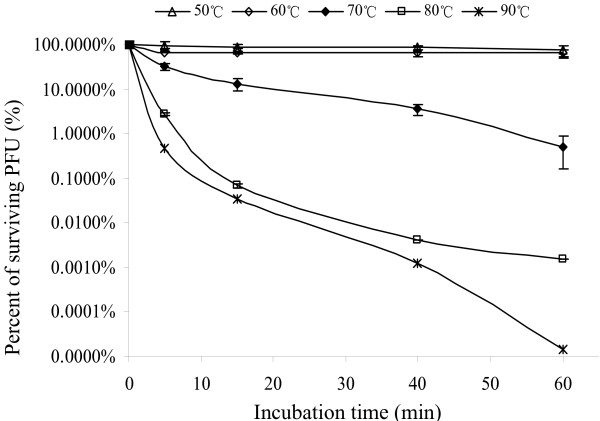
**Thermal stability tests of phage AB1**. Samples were taken at different time intervals to titer the surviving particles and calculate the percentage of infectious phages.

Thermal stability test was carried out to analyze heat resistant capability of phage AB1 at pH6.0. The preliminary experiments showed that phage AB1 stock solution retained almost 100% infection activity after incubation at 37°C for one month (not shown), so higher temperatures of 50°C, 60°C, 70°C, 80°C, and 90°C were chosen to test thermal stability of phage AB1 (Fig. 7). The results showed phage AB1 was extremely heat stable, 73.2% and 64.1% phages still remained alive after 60 minutes incubation at 50°C and 60°C, respectively; only 0.52% phages were alive after 60 minutes incubation at 70°C; while more than 99% phages lost their infection ability in 15 minutes at 80°C, or 5 minutes at 90°C.

### Host range

The susceptibility to phage AB1 was also investigated with four other clinical strains of *A. baumannii*, one clinical strain of *Stenotrophomonas maltophilia*, and other lab bacteria strains such as *Pseudomonas aeruginosa *PAK and PAO1. No strain tested was found susceptible to phage AB1. The results indicated phage AB1 had a narrow host range, consistent with the previous discoveries [18]. Phages specifically targeting *Acinetobacter *spp. have narrow host ranges, usually one host one phage, and it's probably due to the existence of abundant surface bacterial antigens on this bacterium. These antigens are sufficient for different phage recognition [22].

### The susceptibility test

Recently, most clinical isolates of *A. baunannii *were found to be resistant to many antibiotics still in use, making difficult the choice of an adequate antibiotic for the treatment of *A. baunannii *infections [1-3]. In our study, in vitro susceptibility tests of the 5 clinical strains were carried out (table 1). Among the 15 antibiotics tested, all strains were resistant to amoxicillin plus clavulanic acid, ampicillin, cefoxitin, cephalothin, and nitrofurantoin. Strain KD312 was only susceptible to 5 antibiotics, resistant to 10 others including imipenem. Strain KD311, the host of phage AB1, was susceptible to only 6 antibiotics including imipenem but resistant to gentamicin, differing from all other clinical isolates. All these data was coincident with the prevalence of multi-drug resistant *A. baunannii *infections throughout the world, indicating urgent statue of new drugs discovery.

**Table 1:** In vitro susceptibility tests of 5 clinical *A. baumannii *strains

Antibiotics	MIC (μg/ml)^b^									
	
	KD311		KD312		KD331		KD332		KD334	
Amoxicillin/CA^a^	≥32	R	≥32	R	≥32	R	≥32	R	≥32	R
Ampicillin	≥32	R	≥32	R	≥32	R	≥32	R	≥32	R
Cefotaxime	16	I	≥64	R	16	I	16	I	16	I
Cefoxitin	≥32	R	≥32	R	≥32	R	≥32	R	≥32	R
Ceftazidime	≤8	S	≥32	R	≤8	S	≥32	R	16	I
Cephalothin	≥32	R	≥32	R	≥32	R	≥32	R	≥32	R
Gentamicin	≥16	R	1	S	≤0.5	S	2	S	≤0.5	S
Imipenem	≤4	S	≥6	R	≤4	S	≤4	S	≤4	S
Nalidixic Acid	≤16	S	≤16	S	≤16	S	≤16	S	≤16	S
Netilmicin	≤4	S	16	I	≤4	S	≤4	S	≤4	S
Nitrofurantoin	≥128	R	≥128	R	≥128	R	≥128	R	≥128	R
Pefloxacin	≤1	S	≤1	S	≤1	S	≤1	S	≤1	S
Ticarcillin	128	R	128	R	≤16	S	≥256	R	≤16	S
Tobramycin	≤0.5	S	1	S	≤0.5	S	≤0.5	S	≤0.5	S
Trimethoprim/Sulfa	≥320	R	40	S	≤10	S	80	R	≤10	S

a. clavulanic acid at a fixed concentration of 2 μg/ml.

b. R: resistant; S: susceptible; I: intermediate.

## Discussion

Most classified *Acinetobacter *phages are tailed viruses with double stranded DNA genomes. They are classified into three families of the order of *Caudovirales*, including *Myoviridae*, *Podoviridae*, and *Siphoviridae *[18,23]. One exception is phage AP205 which is a ssRNA virus propagating in *Acinetobacter *species [19]. It belongs to *Leviviridae *family and tentatively classified into *Levivius *genus. In this study, phage AB1 had an icosahedral head with a non-contractile tail, and its genome was a molecule of double stranded DNA, so it was tentatively classified as a member of *Siphoviridae *family. Moreover, collar or whisker structures were also observed in the phage AB1 (Fig. 2). Similar complexes have been found in *Escherichia coli *phage T4 [24,25] and lactic acid bacteria phages [26]. These structures are involved in phage assembly, possible regulation functions, sensing environmental conditions, and holding long tail fibers in a retracted conformation [25-30].

Thermal resistant phages were usually isolated from extreme thermal habits [31,32], but they could also be found in other environments. Recently, thermal resistant phages have been isolated and characterized from various dairy products [33,34]. Our experiment results showed that phage AB1 was quite heat resistant, 0.03% phages (1.23 × 10^7 ^PFU/ml) were still infectious even after 15 min incubation at 90°C (Fig. 7). In the preliminary experiments, phage amplification lysate (10^10^-10^11 ^PFU/ml) was heated directly at 100°C for stability test. After 5 minutes boiling, the alive phage concentration was still about 10^5^-10^6 ^PFU/ml (data not shown). Previous studies showed that media components of phage solution could affect the thermal stability of phage [35]. For phage AB1, the lysate supernatant of phage amplification was used directly in thermal stability tests without any additional substance added to LB medium. To demonstrate the mechanism of its notable thermal resistance, more experiments need to be done.

Nowadays, phage therapy has regained much attention due to the emergence of drug resistant pathogens and the dearth of new antibiotics in pipeline. In this study, phage AB1 specific to *A. baumannii *was isolated and characterized. The virus had some outstanding aspects including rapid growth nature, high pH stability, and high thermal resistance. All these characters made this phage very promised for possible applications in eradication of *A. baumannii *contaminations and or treatment of *A. baumannii *infections. However, there was a great diversity of surface antigens existed among the isolated clinical *A. baumannii *strains [22,36,37] and individual phage like AB1 with narrow host range was not suitable to be used directly [38]. In the future, more phages need to be isolated for preparations of cocktails which might be the best choice for phage applications.

## Conclusions

Characterization of phage AB1 showed that it was very efficient in lysing *A. baunannii*, combined with its outstanding thermal stability, it may be a good candidate to be used as an alternative nontoxic green sanitizer. However, host range tests showed phage AB1 did not infect other *A. baunannii *clinical strains included in this study, suggesting that more virulent bacteriophages specific to different *A. baunannii *strains need to be screened and collected in future. A pool of lytic phages might be more useful against *A. baunannii *strains for possible phage applications.

## Materials and methods

### Bacterial strains

This study included a clinical strain of *Stenotrophomonas maltophilia *KD335 and 5 clinical strains of *Acinetobacter calcoaceticus-baumannii *complex, KD311, KD312, KD331, KD332, and KD334. All of them were isolated from hospitalized patients at Tianjin Children's Hospital, Tianjin, P. R. China. Also, other bacteria strains were used in phage host range test, including *Pseudomonas aeruginosa *PAK and PAO1 lab strains.

### Identification of bacterial strains by sequencing the 16s rRNA gene

Clinical strains were confirmed by sequencing the 16s rRNA gene. Supernatant from boiled bacterial cells suspended in distilled water was used directly as PCR templates. Universal primers, 27f (5' AGA GTT TGA TCC TGG CTC AG 3') and 1492r (5' GGT TAC CTT GTT ACG ACT T 3'), were adopted to amplify the 16s rRNA genes [39]. Purified PCR products were sequenced directly with primers. Sequences of 16s rRNA genes were deposited in GenBank under accession numbers FJ871007 (KD311), FJ871004 (KD312), FJ871006 (KD331), FJ871002 (KD332), FJ871003 (KD334), and FJ871005 (KD335).

### Phage enrichment and isolation

Luria-Bertani (LB) broth was used for liquid cultures, and 2% solid agar medium or 0.6% semi-solid agar medium were used for bacteria plating and phage plaque-forming assays, respectively. All incubations were carried out at 35°C. Briefly, identified *A. baumannii *clinical strains were used as indicators for enriching and isolating virulent bacteriophages from marine sediment samples. In brief, marine sediment samples were taken from the coastal seashore (38°59'N, 117°42'E) of China Bohai inner sea. Weighed 5 grams of samples and resuspended in 30 ml LB, 300 μl overnight culture of *A. baumannii *was added to the mixture, incubated at 35°C for 6 hours with shaking to enrich *A. baumannii*-specific bacteriophages. At the end of incubation, drops of chloroform were added to the culture and the flask was left there for 15 minutes without shaking. The culture was filtrated with Whatman filter paper to remove soil particles, and the filtrate was spun down at 11,000 g for 5 minutes to remove bacterial cells and debris. Polyethylene glycol 6000 (PEG 6000) and sodium chloride was added to the supernatant to the final concentrations of 10% and 1 M, respectively. The solution was incubated at 4°C overnight, spun at 11,000 g for 20 minutes. The pellet was dissolved in 1 ml phosphate-buffered saline, the resulting solution was subjected to 0.45 μm filter to remove the residual bacterial cells. The enriched phage solution was mixed with exponential growth culture of *A. baumannii *and plated in semi-solid agar medium after 15 minutes adsorption. Plaques formed on the plates after 4 hours incubation at 35°C. Single plaque was picked out for subsequent phage purification and amplification [40,41].

### Analysis of phage genomic DNA and total phage structural proteins

Molecular manipulations were carried out as previously described [42]. Phage AB1 particles were amplified and purified according to the phage isolation procedures and bacteriophage DNA was isolated by the method described previously [40,41,43]. Restriction endonucleases were used to digest phage genomic DNA, and the genome size was estimated by compilation of DNA fragment sizes resulting from restriction enzymes digestion profiles. DNA molecular standards were from Tiangen Biotech (Beijing) Co., Ltd. To prepare protein sample for SDS-PAGE analysis, purified phage AB1 solution was subjected to Amicon-100 filters, and the phage particles were further washed three times with 0.1 M ammonium acetate solution (pH7.0) to remove possibly existed residual bacterial proteins. Purified phage particles were subjected to SDS-PAGE directly, and the gel stained with Coomassie Blue G-250.

### Morphology study by transmission electron microscope

Phage AB1 solution was filtrated with Amicon-100 filter to remove soluble biological macromolecule fragments of host bacteria. After washing three times with 0.1 M ammonium acetate solution (pH7.0), the retained phage solution was used directly for negative staining as described previously [44]. Photographs were taken with a JEOL1011 transmission electron microscope operating at 100 kV.

### Determination of multiplicity of infection (MOI)

Serial dilutions of bacteriophage stock solution were mixed with the same amount of *A. baumannii *cells. After 15 minutes adsorption, free bacteriophages were removed by centrifugation at 5,000 g for 10 min, pellets were resuspended with LB medium, and samples were taken for bacteriophage titer analysis after 4 hours incubation at 35°C.

### Adsorption rate, latent period, and phage burst size

As described previously [20,21], 10 mM CaCl_2 _was added to the infected culture to measure divalent metal ions effects on adsorption rate of phage AB1, samples were taken at different time intervals to analyze the free phage particles in the solutions with and without addition of calcium ions. One-step growth experiment was carried out according to the previous descriptions [45,46] to determine the latent period and phage burst size. In brief, 50 ml bacterial cells of *A. baumannii *KD311 were incubated to mid-exponential-phase (OD_600 _= 0.4-0.6) and harvested by centrifugation. The pellet was resuspended in 0.5 ml fresh LB medium and mixed with 0.5 ml phage AB1 solution (1 × 10^8 ^PFU/ml). Phage AB1 was allowed to adsorb for 1 min and the mixture was subjected to centrifugation immediately at 13,000 rpm for 30 seconds to remove free phage particles. The pellet was resuspended in 100 ml fresh LB medium and the culture was continuously incubated at 35°C. Samples were taken at 3 min intervals and phage titre was determined by the double-layer-agar plate method. The results were analyzed and the constant phage titer, which represented the number of infective centres, along the latent stage was deduced. The burst size of phage AB1 was calculated by dividing the phage titers at plateau phase by the number of infective centres.

### pH stability and thermal stability test

pH stability and thermal stability tests were carried out as previously described[47,48]. Briefly, certain amount of phage particles were treated under specified conditions. Samples were taken at different time intervals and supernatants from centrifugation were used directly in the assays. Initial phage concentration was about 3.5 × 10^10 ^PFU/ml in LB medium.

### Host range determination

10^8 ^bacterial cells were mixed with melted 0.6% agar (50°C) and this mixture was poured on a 2% solid agar to make double layer agar plates. After solidification, we spotted the isolated bacteriophage stock solution on each plate with different bacterium strain and observed whether lysis plaques emerged.

### The susceptibility test

BioMerieux Vitek 32 system (BioMerieux, Inc., USA) was used in clinical samples diagnosis for bacterial identifications and antibiotics susceptibility tests.

## Authors' contributions

HY designed the experiments and wrote this manuscript; LL performed all phage related experiments; SL analyzed the clinical bacteria strains; HY and SJ supervised the work. The final work was read and accepted by all co-authors.
